# An Analysis of the Optimal Multiobjective Inventory Clustering Decision with Small Quantity and Great Variety Inventory by Applying a DPSO

**DOI:** 10.1155/2014/805879

**Published:** 2014-08-14

**Authors:** Shen-Tsu Wang, Meng-Hua Li

**Affiliations:** ^1^Department of Commerce Automation and Management, National Pingtung Institute of Commerce, No. 51, Min Sheng E. Road, Pingtung 900, Taiwan; ^2^Office of General Affairs, National Taipei University of Nursing and Health Science, Taiwan

## Abstract

When an enterprise has thousands of varieties in its inventory, the use of a single management method could not be a feasible approach. A better way to manage this problem would be to categorise inventory items into several clusters according to inventory decisions and to use different management methods for managing different clusters. The present study applies DPSO (dynamic particle swarm optimisation) to a problem of clustering of inventory items. Without the requirement of prior inventory knowledge, inventory items are automatically clustered into near optimal clustering number. The obtained clustering results should satisfy the inventory objective equation, which consists of different objectives such as total cost, backorder rate, demand relevance, and inventory turnover rate. This study integrates the above four objectives into a multiobjective equation, and inputs the actual inventory items of the enterprise into DPSO. In comparison with other clustering methods, the proposed method can consider different objectives and obtain an overall better solution to obtain better convergence results and inventory decisions.

## 1. Introduction

Stationery discount stores offer a large variety of products in small quantities and have low average profit margins. Moreover, the uncertainty of customer demand causes is a challenge to the stores because product shortages often occur, and backorders are needed [1]. In response to this problem, the stores need to consider relevant inventory decision objectives and develop DPSO (dynamic particle swarm optimisation) for multiobjective planning based on customer demands. The four objectives in inventory decision are total cost, backorder rate, demand relevance, and inventory turnover rate. The understanding of customer demand is a process of integrating dynamic experience, value, situational information, and professional judgment to create and incorporate the information exchange between customers and the stores. Therefore, inventory management decision-making process is important in increasing the store profits. This study applied DPSO in multiobjective planning, and the algorithm considers the disturbance mechanism and tournament selection [2, 3] to improve PSO and assess the multiobjective solution by maximum spread (MS) [4, 5].

According to literature review, few studies simultaneously consider the above four objectives and apply DPSO in the analysis of the customer demand-oriented inventory decision process. By clustering the products and assessing the inventory performances, this study expects to help stationery discount stores to properly control inventory in the face of various customer demands based on inventory knowledge decision making procedures. Moreover, it is expected that sufficient products can be provided at low prices [1–41].

## 2. Literature Review

This section reviews literature on inventory management decisions and multiobjective planning algorithm to analyse the importance of the proposed inventory decision-making process.

### 2.1. Inventory Management Decision

The activity-based costing (ABC) inventory management classification mechanism is the theoretical basis for inventory classification. Ramanathan (2006) [34] argued that the ABC analysis of inventory classification is one of the most widely used approachs of enterprises. Moreover, it is important to consider multicriteria inventory classification and determine weighted linear optimisation. Zhou and Fan (2007) [42] suggested that although the model proposed by Ramanathan (2006) [34] has many advantages, it may inappropriately categorise an item of insignificant criterion but high value as an item in A category. Thus, they proposed an extended R model to provide a more reasonable decision-making method. Hadi-Vencheh (2010) [18] proposed an extended NG-model multicriteria ABC analysis. It is a nonlinear planning model that integrates multicriteria ABC classification, while maintaining the influence of the weights. Chen (2012) [10] suggested that ABC inventory classification is one of the most popular techniques for organisations to efficiently plan and control thousands of inventory items. The proposed approach improves some previous methods by providing a more reasonable and comprehensive performance index and a unique inventory classification without any subjectivity.

In response to the diverse customer demands, different inventory management decision-making concepts have been proposed, such as VMI (vendor management inventory) and RMI (retailer-managed inventory) that can accurately predict inventory and increase inventory turnover rates. Mishra and Raghunathan (2004) [33] pointed out that a successful VMI strategy is the main developmental trend of a coordination strategy and information sharing of the current supply chain management. Moreover, the supply chain management performance can be enhanced by inventory management and relevant cost transfers. They discussed the differences in profitability and compared business performances between VMI and RMI strategies in the retail industry, using the business models of one retailer and two competing brands. They found that when the retailer is faced with two competitors, the business performance and profitability of using the VMI strategy are superior to those using the RMI strategy. Kuk (2004) [24] argued that when VMI strategy is implemented in the electronic industry, the information technology implementation barrier and mutual trust between vendor and retailer are the main factors affecting the success of the VMI strategy. Dong and Xu (2002) [16] suggested that inventory management proprietary rights belong to the vendor. Moreover, when applying the VMI inventory management strategy, the suppliers bear the inventory costs originally borne by the retailer. According to Yao et al. (2007) [43], the inventory management proprietary rights are held by the retailer; hence, the retailer should assume inventory-related costs. The upper, middle, and lower suppliers of the supply chain management share information and communicate, while the retailers can reduce inventory purchase costs. The suppliers can change the inventory level and minimise inventory costs to enhance the business performance of the supply chain as a whole. Kannan et al. (2013) [22] analysed two cases concerning the benefits that a VMI agreement could bring for the one-supplier multiple-customer case: a supply chain managed in a traditional manner and VMI when both the vendor and the customers belong to the same organisation. The analysis is based on the economic ordering quantity (EOQ) formula and its related total cost. The novelty is captured by evaluating one vendor, multiple buyers, and multiple product situations.

Backorder rates and demand relevance have shown the important objectives of the enterprise when satisfying a set level of customer service, and many scholars have applied various methods to meet such customer service levels. Axsäter (2003) [6] pointed out that, through one-way horizontal allocation and alternative questioning, supply chain management performance is measured by service level. Lee et al. (2007) [25] studied the effective horizontal allocation of the supply chain to promote the overall service level for different groups of customers. Hsu and Tsou (2010) [1] argued that inventory management is very important to an enterprise, and its purpose is to use the least cost to maintain a high standard of service, thus, reducing the likelihood of backorders and meet customers' demands in products. How to trade off such conflicting objectives is a challenge of multiobjective inventory control. They thus extended the three-objective inventory control model under backorder, as proposed by Agrell (1995), to the case of sales loss by adding local search and the hybrid multiobjective particle group optimisation of clustering mechanism to solve the problem of inventory control under different models. The results were compared with the robust Plato evolutionary algorithm. It was found that the nondominated solution of the hybrid multiobjective PSO optimisation is significantly better than the robust Plato evolutionary algorithm, under three performance measurement indicators. Finally, the solutions of different inventory models, in the cases of backorder and sales loss, were compared. For sales loss, enterprises are particularly concerned about sales loss due to backorders; thus they pay special attention to inventory management by keeping track of the sales condition, ordering appropriate inventory, and reducing inventory costs.

### 2.2. Multiobjective Planning Algorithm

Multiobjective planning is to determine a better solution from multiple objectives. Many scholars have applied algorithms in the solution of multiobjective problems to obtain more efficient mechanisms. Liu et al. (2007) [29] proposed a method containing a synchronous local search and a new particle updating method. The synchronous local search can directly implement local fine-tuning to enhance the global search capability of PSO, thus, solving premature convergence and maintaining solution diversity for a fuzzy global-best solution. Hsieh et al. (2007) [19] proposed a method to delete excessive similar particles in the external scratchpad through cluster operation. The circle centre dominated method was adopted to determine the global optimal solution. The concept of gas diffusion allows each particle to have a footing to expand externally. The specific method depends on the currently stored nondominated solution to calculate an imaginary circle centre. The coordinates of the circle centre are composed of the maximum values of the target functions in the current storage space. Krichen et al. (2012) [23] applied a number of linear target functions of multiobjective linear optimisation problems (MOLPs) to optimise a convex polyhedron. They proposed a new method to generate a set of effective result MOLPs space, which is based on the adjacent concept of highly efficient extreme points. The mining method can generate highly efficient extreme points and viewpoints of maximum efficiency. Moreover, a highly efficient combination of adjacent extreme points of defined borders was proposed.

For inventory multiobjective decision-making, Wang and Shu (2007) [41] developed a fuzzy decision making model to assess the overall performance of new product supply chain design, thus minimising the total supply chain costs and maximising the value. A genetic algorithm was used to obtain the optimal solution. Özgen et al. (2008) [4] integrated the analytic hierarchy process (AHP) method and multiobjective possibility planning and developed an application model of supplier selection and order allocation problems, that covered qualitative and quantitative factors. That model aimed to minimise the total purchase cost and total rejections and maximise the weighted supplier points. Mansouri et al. (2012) [31] proposed the optimisation of multiple objectives to establish order supply chain management. They reviewed build-to-order supply chains as the decision support tool of multiobjective optimisation key technology. From the perspective of multiobjective optimisation of the existing optimisation model, their method attempted to develop relevant decision support by considering the different interests of each supply chain, without involving manufacturers. Hence, service-based objectives were developed to effectively satisfy the requirements of the objectives. Bouchery et al. (2012) [7] explored the multiobjective problems. Using the amended EOQ model, they studied sustainable order quantity and analysed the characteristics of a highly efficient solution set (Pareto optimal solution). These results were used to provide regulatory policies of different opinions in the control of carbon emissions. They provided an interactive process to allow decision makers to quickly identify the optimal solution of the solution set. The proposed interactive process is a new combination of multicriteria decision analysis technique.

## 3. Research Method

### 3.1. Description of Single-Objective Function

This paper considers a number of commonly used inventory management objectives, including total cost, backorder rate, demand relevance, and inventory turnover rate, which are elaborated in detail, as follows [9, 14, 26, 34].

#### 3.1.1. Total Cost Target Equation

Symbols:

 TC
: Total cost
 
*b*_*i*_: Item *i* set-up cost
 
*T*_*j*_: Cluster* j* order cycle
 
*D*_*i*_: Demand in unit time of item *i*
 
*h*_*i*_: Holding cost of each unit of inventory in unit time of item *i*
 
*U*_*j*_: Item sets contained in cluster *j*
 
*N*: Maximum number of clusters
 
*x*_*id*_: Current position of particle *i* in dimension *d*
 
*z*: Integral value in the range from 2 to *N*
 
*B*_*m*_: Major purchase cost of issuing order to supplier *m*
 
*E*_*j*_: Supplier sets contained in cluster *j*.

As updating of the breakpoint needs to comply with *x*_1,2_ ≤ *x*_2,3_ ≤ ⋯≤*x*_(*N*−2,*N*−1)_ ≤ *x*_(*N*−1,*N*)_, this paper uses another method of improvement, namely, if *x*_(*z*−2,*z*−1)_ > *x*_(*z*−1,*z*)_, then *x*_(*z*−2,*z*−1)_ = *x*_(*z*−1,*z*)_. The total cost consists of ordering and item holding costs. The cost computation equation of direct clustering, as proposed by Chakravarty (1985) [9], is used. As the number of suppliers is considerably high, and orders are issued according to the suppliers, the items in clusters are provided by a number of different suppliers. Therefore, the orders are sent out separately. The cost target equation should consider the situation of a cluster consisting of multiple suppliers, as follows [9, 14, 26, 34]:

(1) $$\begin{array}{c} T_{j}=\sqrt{\frac{2\sum_{i\in U_{j}}D_{i}\left(\sum_{i\in U_{j}}b_{i}+\sum_{m\in E_{j}}B_{m}\right)}{\sum_{i\in U_{j}}D_{i}h_{i}}}, \end{array}$$

(2) $$\begin{array}{c} \text{TC}=\overset{N}{\underset{j=1}{\sum }}\left[\frac{\sum_{i\in U_{j}}D_{i}\left(\sum_{i\in U_{j}}b_{i}+\sum_{m\in E_{j}}B_{m}\right)}{T_{j}}+\frac{1}{2}T_{j}\underset{i\in U_{j}}{\sum }D_{i}h_{i}\right]. \end{array}$$

For an algorithm with cost as the target equation, it only needs to input the clustering results into (2) when estimating the particle fitness value. The purpose is to minimise additional ordering and item holding costs.

#### 3.1.2. Backorder Rate

The case in this study is a stationery discount store, which is characterised by products of small quantity and great variety, varying customer demands, and frequent backorders. The frequent backorders lower the customer demand because customers may have lost patience and turned to other stores. This is consistent with the practical situation [9, 14, 26, 34].

In the model construction process of this study, a known and fixed parameter *γ* is added, which represents the proportion of decrease in demand during the backorder period, and 0 ≤ *γ* ≤ 1. The rate of change in demand during the backorder period (i.e., the demand in unit time) is defined, where, *f*(*t*) is the unit time demand rate during the backorder period, *D*_*i*_ is the fixed demand rate of item *i*, *T*_*j*_ = *t*_1_ + *t*_2_, *t*_1_ is the time of depletion of item *i*, and *t*_2_ is out-of-stock time of item* i*. A decreasing function relating to demand and time is developed.

Backorders occur in the period of *t*_2_, and the demand rate in period *t*_2_ is the decreasing function of out-of-stock time. This paper defines *f*(*t*) = *D*_*i*_(1 − *γt*) and (1 − *γt*) ≥ 0, which is the change in demand (i.e., the slope). Hence, by the integration of *f*(*t*), we can obtain the function of the curve. The *t*_2_ value is inputted to obtain the maximum order level, as shown below:

(3) $$\begin{array}{c} \overset{t_{2}}{\underset{0}{\int }}f\left(t\right)dt=\overset{t_{2}}{\underset{0}{\int }}D_{i}\left(1-\gamma t\right)dt=D_{i}t_{2}-\frac{D_{i}\gamma \cdot t_{2}^{2}}{2}. \end{array}$$

We assume *c*(*t*) = *D*_*i*_ *t* − (*D*_*i*_ *γ* · *t* ^2^/2) is the out-of-stock function of period *t*_2_ and conduct integration of *c*(*t*) to obtain the area of out-of-stock in period *t*_2_ (i.e., the total number of backorders), as shown below:

(4) $$\begin{array}{c} \overset{t_{2}}{\underset{0}{\int }}c\left(t\right)dt=\overset{t_{2}}{\underset{0}{\int }}\left(D_{i}t-\frac{D_{i}\gamma \cdot t^{2}}{2}\right)dt=\frac{D_{i}t_{2}^{2}}{2}-\frac{D_{i}\gamma \cdot t_{2}^{3}}{6}. \end{array}$$

Hence, we can calculate the average backorder level of Item* i *(*L*_*i*_) and the order level of Cluster* j* (*L*_*j*_), as follows:

(5) Li=(Dit222−Diγ·t236)·1t1+t2=Dit222(t1+t2)−Diγ·t236(t1+t2),

(6) $$\begin{array}{c} L_{j}=\frac{\sum_{i\in U_{j}}L_{i}}{i}. \end{array}$$

#### 3.1.3. Demand Relevance

The target equation depends on the demand relevance of items. For example, when purchasing a mechanical pencil, the refill or rubber is often purchased at the same time. In other words, before applying the algorithm, the demand relevance of items should be understood. This paper employs the relevant items of the stationery discount store to compute the demand relevance [9, 14, 26, 34].

Below are the steps for the computation of item relevance:

(1) Calculate the demand proportion of each final product.
(2) According to the bill of material of each final product, calculate the number of various items to produce a final product.
(3) Multiply the product demand proportion with the number of items obtained in Step (2).
(4) Sum up the quantity of the same items used in each type of item, and calculate the total demand of each item.
(5) Convert the total demand of items into the demand relevance data of a two-dimensional matrix.
(6) Standardise the two-dimensional demand relevance data to render all values in the range of 0 to 1, and the standardisation equation is as follows:
  (7) $$\begin{array}{c} R_{is}=\frac{W_{is}-W_{\min}}{W_{\max}-W_{\min}}, \end{array}$$
 where, *R*_*is*_: the demand relevance of item *i* and item *s* after standardisation; *W*_*is*_: the demand relevance of item *i* and item *s* before standardisation; *W*_max⁡_: maximum demand relevance before standardisation; *W*_min⁡_: minimum demand relevance before standardisation.

By following the above steps, we can calculate the demand relevance of the items, which is relative between the items, such as item *i* against item *s* and item *s* against item *i*. Hence, we only need to consider one situation in the computation of total relevance. When applying the algorithm, items classified in the same cluster can be sorted by item number in an ascending order. The demand relevance of small-numbered items is summed against big-numbered items, so that the total demand relevance of each cluster can be obtained. Finally, the total demand relevance of each cluster is summed to obtain the total demand relevance of the clustering results. The total demand relevance is the fitness value of the particle, and the objective of the target equation is to maximise the total relevance of each cluster.

#### 3.1.4. Inventory Turnover Rate

The inventory turnover rate refers to the rate of turnovers of inventory items in a certain period. In general, enterprises use the inventory turnover rate as the indictor of inventory management [9, 14, 26, 34], as shown in (9)

(8) Inventory  turnover  rate    =    (sales  cost  within  the  cycle⁡)(average  cost  within  the  cycle⁡)  =∑i=1VDi∑i=1VJi,

where, *D*_*i*_: demand for item *i*, *J*_*i*_: inventory of item *i*, and *V*: volume of inventory items.

Average inventory volume of cluster* j *= ∑_*i*∈*U*_*j*__(*D*_*i*_ *T*_*j*_/2).

Inventory volume within the cycle = ∑_*i*=1_^*V*^ *J*_*i*_ = ∑_*j*=1_^*N*^∑_*i*∈*U*_*j*__(*D*_*i*_ *T*_*j*_/2)

(9) $$\begin{array}{c} \text{Inventory}\;\text{turnover}\;\text{rate}=\frac{\sum_{i=1}^{V}D_{i}}{\sum_{j=1}^{N}\sum_{i\in U_{j}}\left(D_{i}T_{j}/2\right)}. \end{array}$$

Equation (9) is the computation equation of the target equation inventory turnover rate, where the objective is to maximise the inventory turnover rate of the clustering results.

### 3.2. Descriptions of Standardisation of Multiobjective Target Equation

In this section, the single-objective target equations are combined using the objective planning method to obtain the optimal clustering results that can satisfy various target equations. Since the units of the objectives vary, the objectives are standardised using a simple standardisation method to convert the target value of each objective into a value in the range of 0 to 1. The standardisation method assesses the effect measure of the clustered target values and unclustered target values. The clustered target value is the target value calculated by inputting the clustered items into each single-objective target equation. The unclustered target value is the target value calculated by inputting the items classified as a cluster into the target equation.

The dummy standard sequence consists of the optimal *s*_*ij*_ of the assessment result of each attribute. This optimal result is determined by the objective of maximisation or minimisation of the attribute. The effect measure *r*_*ij*_ represents the relationship of each element of sequence {*s*_*i*1_, *s*_*i*2_,…, *s*_*in*_} corresponding to attribute *a*_*i*_ and the dummy standard sequence. The calculation of effect measure can distinguish upper effect and lower effect measures, according to the maximisation or minimisation target of the attribute, as illustrated below [11].

The upper effect measure: applicable to attributes with the objective of maximisation (i.e., the larger the better), such as inventory turnover rate. Hence, the maximum result *u*_*i*_^max⁡^ of all programs of attribute *a*_*i*_ is used as the element corresponding to the dummy standard sequence. The definition of the upper effect measure is as follows:

(10) $$\begin{array}{c} r_{ij}=\frac{s_{ij}}{u_{i}^{\max}},\;\text{where}\;u_{i}^{\max}=\underset{j}{\mathrm{Max}}\;s_{ij}. \end{array}$$

The lower effect measure: applicable to attributes with the objective of minimisation (i.e., the smaller the better), such as total cost. Hence, the minimum result *u*_*i*_^min⁡^ of all the programs under attribute *a*_*i*_ is used as the element corresponding to the dummy standard sequence. The lower effect measure is defined, as follows:

(11) $$\begin{array}{c} r_{ij}=\frac{u_{i}^{\min}}{s_{ij}},\;\text{where}\;u_{i}^{\min}=\underset{j}{\mathrm{Min}}\;s_{ij}. \end{array}$$

As defined above, the value of effect measure *r*_*ij*_ is in the range between 0 and 1. The greater the value, the better the effect of program *b*_*j*_ under attribute *a*_*i*_.

### 3.3. Pareto Set Method to Solve the Nonlinear Problem of Target Equation

Regarding the nonliner problem in Section 3.1.2, this paper adopts the Pareto set method (Lin et al., 2011) [28], which can be divided into two parts. Steps (1)–(5) are the first part. The initial approximation of the Pareto set is to obtain a linear solution to approximate the nonlinear problem for *β*-Pareto prediction in the second part. The approximation method in the first part adopts an appropriate number of line segments in order to approximate the original nonlinear Pareto set until the approximation error is within the acceptable range. Then, the feature points upon approximation are used as the linearisation of the nonlinear constraint in the second part to generate a group of appropriate linear constraints. The details of the method are described below [12, 33, 36].

(1) The nonlinear Pareto set is indifferent to reliability and is regarded as the start of the initial Pareto set. If the* j*th target function is used as the independent variable, another function value is the dependent variable. First, we calculate the optimal solution of *f*_*j*_, with *f*_*j*_(*x*_j_*) as the lower bound of independent variable $\underline{f}$, as shown in (12)

(12) min⁡x fj(x),s.t.  g(x)≤0.

Next, after computing the optimal solution of another target function *f*_*j*_(*x*_j_*), we input the optimal solution into target function *f*_*j*_(*x*_j_*) as the upper bound of independent variable $\bar{f}$. Therefore, after computing the lower and upper bounds of the independent variable, we can obtain the Pareto set function using *p*(*f*), $\underline{f}\leq \varepsilon_{j}\leq \bar{f}$, as shown below. With the upper and lower bounds as the starting points; proceed to Step (2):

(13) $$\begin{array}{c} p\left(f\right)=p\left(\varepsilon_{j}\right):\left[\begin{array}{c} \underset{x}{\min}\begin{array}{cc} & f_{l}\left(x\right) \end{array}\\ \text{s}.\text{t}.\begin{array}{cc} & g\left(x\right)\leq 0 \end{array}\\ f_{j}\left(x\right)\leq \varepsilon_{j},\forall j=1,2,j\neq l \end{array}\right]. \end{array}$$

(2) After the computation of the lower and upper bound functions, as shown in (12) and (13), we then calculate the error between the lower and upper bounds to enter Step (3).

The block sandwich squeezing method uses the linear function of the upper and lower bounds to squeeze the original function until the error of the lower and upper bounds is smaller than expected. If the Pareto set is regarded as a convex function *p*(*f*), in other words, *ε* of the restraint method is used as the independent variable *f* and the value of another target function of the nondominated solution as the dependent variable *p*(*f*), we can produce a group of linear functions of the upper and lower bounds. The restraint method can only be used in the appropriate range. If there are *b* cut-off points in the range of $\left[\underline{f},\bar{f}\right]$, the Pareto set can be divided into *b* + 1 blocks for discussion, *S*_*b*_ = {*f*_*i*_, *i* = 0,1, 2,…, *b*}, $f_{0}=\underline{f}$; then, the upper bound function *u*_*i*_(*f*) can be written as:

(14) u(f):ui(f)=p(fi)+p(fi+1)−p(fi)fi+1−fi(f−fi),f∈[fi,fi+1].

The upper bound function is moved downward until the upper bound function and nonlinear function intersect at point *f*_*i*_′. The process of finding *f*_*i*_′ is a single-objective optimisation problem, as shown below:

(15) min⁡f p(f)−eif,s.t.  fi≤f≤fi+1,where  ei=p(fi+1)−p(fi)fi+1−fi.

If *f*_*i*_′ is the optimal point to produce the optimal solution to target (15), we can present the lower bound function *l*_*i*_(*f*) as follows:

(16) $$\begin{array}{c} l\left(f\right):l_{i}\left(f\right)=p\left(f_{i}^{\prime }\right)+\frac{p\left(f_{i+1}\right)-p\left(f_{i}\right)}{f_{i+1}-f_{i}}\left(f-f_{i}^{\prime }\right). \end{array}$$

As the range of lower bound function *l*_*i*_ can be obtained by considering the following segment *l*_*i*+1_ and previous segment *l*_*i*−1_, the intersection point of *l*_*i*_ and *l*_*i*+1_ can be computed, using *f*_*i*_^*l*^ to obtain the upper bound of *l*_*i*_ and the interaction point *f*_*i*+1_^*l*^of *l*_*i*_ and *l*_*i*−1_ to obtain the lower bound of *l*_*i*_. Given that the cut-off point is appropriate, we can approximate the original function by the lower bound and upper bound linear functions, as follows:

(17) $$\begin{array}{c} l\left(f\right)\leq p\left(f\right)\leq u\left(f\right),\;f\in \left[\underline{f},\bar{f}\right]. \end{array}$$

(3) Check whether the final error, as obtained in Step (2), is smaller than the one acceptable to the decision maker. If it is smaller, the first part is complete, and it enters Step (5); if not, enter Step (4).

(4) Using *f*′ in the calculation of the lower bound function in Step (2) as the cut-off point, increase the number of cut-off points of the initial Pareto set in order to use more line segments for approximation. According to the modified Sandwich squeezing method, as proposed by Tan et al. (2006) [36], there will be more cut-off points in place of a greater Pareto set curvature. Then, enter Step (2).

(5) After finding the appropriate linear model of the Pareto set in the target space, a group of corresponding restraint conditions in the design space is determined. In other words, the cut-off point as the “feature point” is used to determine the corresponding solution in the design space and conduct the linearisation of the first order Taylor function of the active restraint conditions. A group of restraint conditions is obtained, then enter Step (6).

(6) The initial reliability, as determined by the decision-maker, is used to horizontally move the restraint conditions; then, enter Step (7).

(7) The Pareto set method for linear problems is used to calculate the Pareto set from the initial reliability to the final reliability; then, enter (8).

(8) The horizontal movement of the linear Pareto set and the movement of the nonlinear Pareto set should have an error, which is caused by the nonlinear degree. The error is checked by the distance between the optimal points of the same single objective in the target space of the two problems; after computation, enter Step (9).

(9) Check whether the error calculated in Step (8) is acceptable to the decision-maker, if it is acceptable, the implementation of the method is ended; otherwise, enter Step (10).

(10) In theory, using infinite number of line segments to approximate the nonlinear function can have the characteristics of the function. Hence, error *ε* acceptable to the decision maker can be narrowed by half to enter into the first part to restart this method.

### 3.4. DPSO (Dynamic Particle Group Optimisation)

The DPSO algorithm procedure is as shown below [8, 20, 25].

1. To establish and initialise a few subgroups *I*_*g*_  (*g* = 2,3, ..., *G*), *G* is the maximum number of groups.
2. In *m* times of implementation (*m* is determined by the number of particle groups and dimensions), after calculating the fitness value of the particles of the subgroups, disturb the particles when updating the particle position.
3. In the *m*th implementation, after calculating the particle fitness value of various subgroups, use the modified velocity equation to update the position of each particle by referring to the position of the particles of different subgroups.
4. Consider the changed fitness values of particles of the optimal solution and implement the tournament selection.
5. After the convergence of all particles in a certain subgroup, use the fuzzy* c* mean method to adjust the cluster centre.

**Figure 1:**
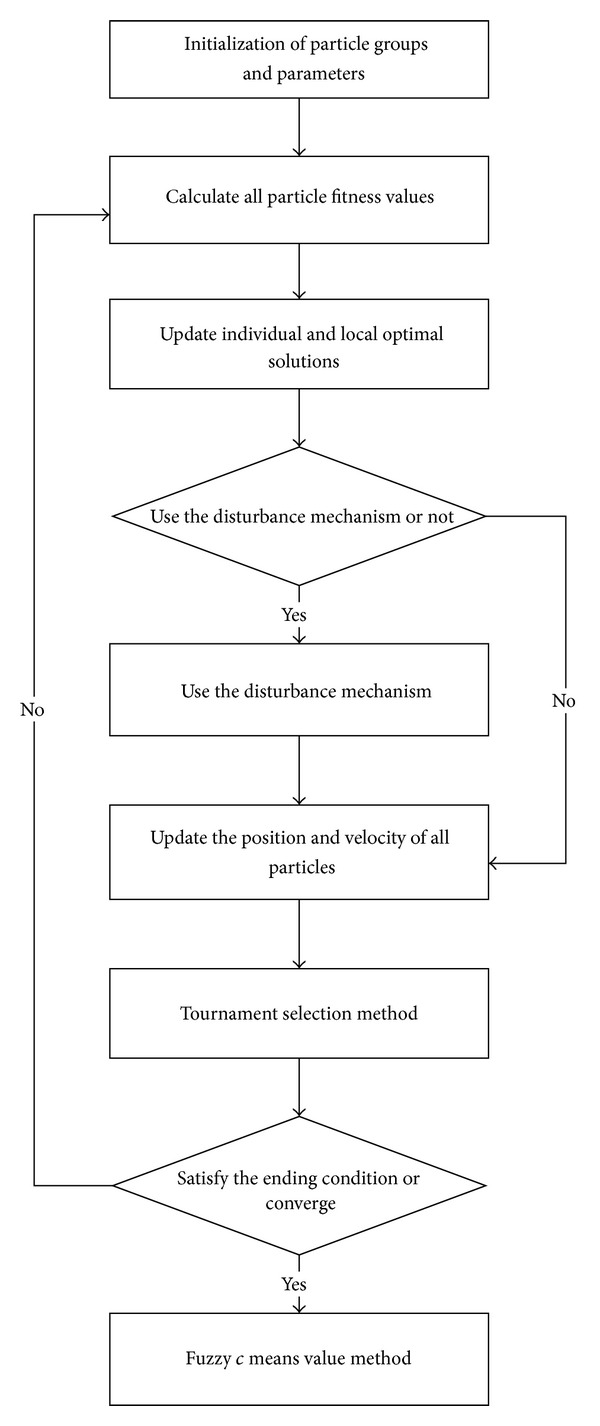
DPSO procedure.

The algorithm procedure is as shown in Figure 1.

The entire particle group set (*I*) is divided into a number of subgroups according to the number of groups (*I*_*g*_). In the given number of groups, we use the disturbance mechanism in the subgroups to test different combinations of group centres, thereby increasing particle diversity. The second step compares the fitness values generated by the different groups and uses the modified velocity equation to update particle action. The third part uses the fuzzy theory to consider the overall distribution of data to obtain the optimal centre when particles converge.

#### 3.4.1. Disturbance Mechanism

The PSO evolution indicates that particles will move to a known location according to previous experience, which is characterised by fast convergence. Hence, after iteration, many particles tend to be attracted to the cluster in certain areas, resulting in stagnation of late iteration. Although the particles have very good search capabilities in some local areas, they cannot find a better solution outside these areas. In other words, these particles cannot escape the local optimal solution and are unable to determine the global optimal solution. This paper adds a disturbance mechanism in the moving process Tsai et al. (2010) [38] in order to increase the opportunity for the particles to escape the local optimal solution. Disturbance is a mechanism similar to genetic mutation for optimising poorer dimensional value. The design of the disturbance mechanism is a random disturbance within the boundaries but without causing the problem of escaping the spatial boundary. The operational process of the disturbance mechanism for the number of particle groups of *G* and problem dimension at *k* is elaborated as follows.

*N* is defined as the multiplication of DR (disturbance rate) and *G*  (*N* = DR × *G*). We randomly select *n* dimensions of *L* particles to add noise for disturbance. The selected dimension is between 1 and *k*  (1 < *n* < *k*). The disturbance noise is between 0 and the gap between the boundary and the dimension value. In late iterations, most particles start to concentrate around the optimal position of some regions. To prevent the local search of particles and ensure efficient search of the optimal solution, the disturbance amount is increased. Hence, to satisfy the above-mentioned requirements, the disturbance probability increases with the iteration number in a linear manner. The DR, as set in this paper, linearly increases from 0.06 to 0.28. The disturbance mechanism will cause the particles to move in the direction of an unknown space in the selected dimension, rather than upon previous experience, guide the particles from falling into a local optimal solution, and encourage particles to move in directions that have not been explored. The disturbance operational virtual codes are as shown below. Random parameter *R* is Gaussian noise (mean value 0.5, standard deviation 0.9), where *R* is a random value between 0 and 1; if *R* < 0.5, it is a low value disturbance, otherwise, it is a high value disturbance. After determining the direction, the disturbance value is determined by multiplying a random value rand in the range of [0, 1], where *l*(*y*_*i*,*j*_) and *u*(*y*_*i*,*j*_) are the lower bound and upper bound searches of *y*_*i*,*j*_, respectively.

 If *R*< 0.5
 
*y*_*i*,*j*_ = *y*_*i*,*j*_ − [*y*_*i*,*j*_ − *l*(*y*_*i*,*j*_)] × rand()
 Else
 
*y*_*i*,*j*_ = *y*_*i*,*j*_ − [*u*(*y*_*i*,*j*_) − *y*_*i*,*j*_] × rand()
 End.

#### 3.4.2. Tournament Selection

The global optimal solution (*G*_best_) of the PSO algorithm has the greatest impact on the movement direction of the entire cluster. It is mentioned above that the main difference of single-objective optimisation and multiobjective optimisation is the solution. In single-objective optimisation, there is only one solution, that is, the global optimal solution; however, in multiobjective optimisation, objectives trade off each other and there are generally more than two optimal solutions. Thus, the selection of the global optimal solution will seriously affect the convergence of the algorithm and solution dispersion. Hence, the global optimal solution of a multiobjective optimisation problem is redefined. The global optimal solution of single-objective optimisation is to select the only optimal solution in the evolutionary process. However, this definition is not applicable in a multiobjective problem. How to select the global optimal solution from the nondominated solution set to provide a guide for the subsequent evolution is an important issue.

By referring to the binary tournament selection (M. Chakraborty and U. K. Chakraborty, 1997) [2], this study proposes the tournament selection of the global optimal solution. Literally, it selects the winner by tournament, as a competitor that wins over all other rivals. The winning competitor is the result determined using the mechanism. In multiobjective optimisation, the global optimal solution assigned to each particle may vary, meaning there are different leaders. The mechanism expects, in the evolution process, to effectively distribute the global optimal solution and move forward in the direction of a less dense solution space, in order to find more nondominated solutions that enhance solution diversity without delaying the convergence velocity of the algorithm.

**Figure 2:**
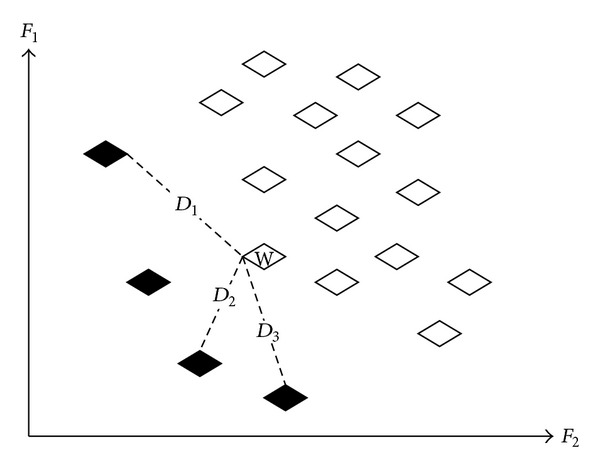
Tournament selection.

The tournament mechanism selection is as shown in Figure 2. Each diamond represents a particle; a dotted gray diamond represents the elite particle of an external register; a solid white diamond represents a particle in evolution; and *d* is the distance between the evolving particle and the particle in the external register. The process to define the global optimal solution of particles in evolution is described in the following three steps.

1. Compute Euclidean distance *d* between the particle in evolution and the particle in the external register.
2. Regarding each particle in evolution, from all the distances of the particle and the particles in the external register, randomly select three distances and three particles of optimal experimental effect; the three distances comprise the competition among three competitors.
3. Finally, compare the three distances, the one with the smallest distance corresponds to the particle of the external register; namely, the particle is assigned as a leading direction of evolution for *G*_best_.

*D*_*i*,*j*_ is computed as follows, where *ox*_*i*_ denotes the *i*th particle in the external register and *ov*_*j*_ denote the *j*th particle in evolution:

(18) $$\begin{array}{c} D_{i,j}^{}=\sqrt{{\left[F_{1}(ox_{i})-F_{1}\left(ov_{j}\right)\right]}^{2}+{\left[F_{2}(ox_{i})-F_{2}\left(ov_{j}\right)\right]}^{2}}. \end{array}$$

As shown in Figure 2, in the case of Particle *W* in evolution, if we randomly select three distances from all the distances between the particles of all particles of the external register (*D*_1_,* D*_2_, and* D*_3_),* D*_2_ is apparently shorter than the remaining two distances. Therefore, the nondominated solution is assigned as the global optimal solution (*G*_best_) of Particle* W*. As the three distances are randomly selected, each particle in the external register has the opportunity to be assigned as the global optimal solution of the particle in evolution, thus enhancing solution diversity and reducing the local concentration of all particles caused by some particles leading in the external register, where there are too many particles in evolution. In this way, many particles in the local optimal position can be limited, and particle diversity is reduced.

### 3.5. Multiobjective Solution's Evaluation Method-MS

The difference between the single-objective optimisation problem and the multiobjective optimisation problem is that the optimal solution to the former problem is a point in the solution space. Therefore, a comparison of the single dimension value of the solution space is needed to distinguish the quality of the solution. However, in the latter case, as the relationship between optimal solutions is not dominating, the solution space has a linear or curvature distribution for multiple groups of solution sets. Hence, algorithm efficiency should be evaluated using multiobjective assessment. The measurement and evaluation standards used in this paper are elaborated as follows.

The difference performance indicator cannot be used to discuss the similarities of the boundary or the Pareto front, but can comprehensively evaluate the similarities between combination uniformity and the optimal Pareto front. Hence, MS is used to evaluate the similarities of the boundary and Pareto front of the algorithm [4, 5], MS evaluation parameters are as follows:

(19) $$\begin{array}{c} \text{MS}=\sqrt{\frac{1}{n}{\overset{n}{\underset{i=1}{\sum }}\left[\frac{\min\left(s_{i}^{\max},S_{i}^{\max}\right)-\max\left(s_{i}^{\min},S_{i}^{\min}\right)}{S_{i}^{\max}-S_{i}^{\min}}\right]}^{2}}. \end{array}$$

#### 3.5.1. MS = 1

In (19), as MS is the root of a square, it is definitely positive. However, among the MS evaluation parameters, *S* denotes the optimal solution, and *s* denotes the identified solution. The max and min denote the minimum or maximum value of* S* or *s* in the *i*th dimension, indicating the number of targets to be optimised. Moreover, when the optimal solution and the solution found at the boundary are the same, the sum will be the same. In other words, when MS is 1, the extensibility of the found solution at the boundary will be perfect.

#### 3.5.2. MS < 1

In general, MS is almost below 1, when MS is below 1, it means that the solution distribution in the evaluated dimension covers the value of the Pareto front in this dimension.

#### 3.5.3. MS > 1

As the MS assessment parameter is the root of the square of the assessment parameter of the extensibility in various dimensions, in particular situations, MS can be above 1, suggesting that the solution distribution has not covered the value of the Pareto front in this dimension. In other words, the difference between the distribution of a searched solution and the Pareto front is great.

## 4. Case Verification

Using DPSO, this study develops a clustering method for inventory items and compares it with other common clustering methods in order to verify the effectiveness of the proposed method. Other common clustering methods include the multiobjective PSO clustering algorithm and the ABC analysis method, where each item is an independent group and all the items are classified as a group (namely, no clustering). The cost target equation calculates the total sum of ordering costs and inventory holding costs. A smaller cost means better clustering results. In Table 1, “DPSO” is the application of DPSO, “PSO” is the multiobjective PSO clustering algorithm, “ABC” is the ABC analysis method, and “G” is the clustering method of independent groups.

### 4.1. Comparison of Multiobjective Target Value and Single-Objective Target Value

In the case study, this paper refers to nearly three years of monthly inventory data, covering 6938 items and 156 suppliers. The data are consistent with the classification criteria of the ABC analysis method. Table 1 lists the target values obtained using various clustering methods in the case of a different single-objective target equation. As shown in Table 1, the proposed DPSO algorithm is superior to other clustering methods.

If the store uses the clustering method, the annual cost of inventory items is 2,635,849.67 NTD. If the store uses the proposed DPSO method, it can save 326,580.55 NTD annually, with a saving of 10.9%. The comparison with other clustering methods is as shown in Table 2. The computation of the percentage in Table 2 is as shown below:

(20) $$\begin{array}{c} \text{DPSO}:P\;=\;\frac{\left(\text{cost}\;\text{savings}\right)}{\left(\text{PSO}\;\text{clustering}\;\text{method}\right)}. \end{array}$$

The results of applying the proposed DPSO method in actual inventory data are as shown in Tables 3 and 4. Although the clustering results using the proposed DPSO method are better than the results of the single-objective methods, the ranking of the multiobjective method is almost second in terms of individual objective comparison. The ranking of the backorder rate is the highest. The percentage difference computation of Tables 3 and 4 is as shown below:

(21) Percentage  difference=(multiobjective  target  value−single-objective  target  value)(multi-objective  target  value).

### 4.2. Comparison of Different Multiobjective Algorithms

Deb et al. (2002) [14] proposed the nondominated sorting genetic algorithm II (NSGA-II), which is elite and definitely maintains the difference mechanism. The offspring generation sized *N* is produced and combined with the parent generation sized* N*. In the ranking of the nondominated solutions, *N* individuals of better quality are retained to ensure the optimal nondominated solutions of the parent and offspring generations. In addition, crowded tournament selection is applied to ensure the difference between solutions. However, if the number of nondominated solutions is fewer than the overall clusters in the first search, besides the solution close to Parent front, other solutions that approximate the Pareto front will be retained to affect the convergence of the algorithm. This paper compares DPSO, PSO, and NSGA-II to confirm that using a disturbance mechanism and tournament selection to select global optimal solution can achieve better convergence effect.

Each test function experiment is performed 30 times. The experimental test function is the multiobjective function introduced in Section 3.1, the fitness evaluation assessment scale (FEAS) is 20,000, the initial number of cluster sizes (or number of chromosomes) is 120, and the number of new particles (chromosomes) generated in each iteration (movement) is 80. The simulation results are obtained after repeating the Monte Carlo experiments 30 times for each algorithm. Parameter setup of the Pareto archived evolution strategy (PAES) and nondominated sorting harmony search (NSHS) are shown in Table 6. Tables 5 and 6 show the settings of the various algorithm parameters.

Moreover, in order to evaluate and compare the localisation accuracy of the four approaches, we use the normalised localisation error (NLE) proposed by Manjarres et al. (2013) [30].  Table 7 lists the algorithm for each scenario and the average means, minimum and standard deviation of the NLE values associated to the topologies belonging to the approximated Pareto front after 30 Monte Carlo experiments [14, 39].

This paper adopts the solution spread measure (SSM), ratio of nondominated individuals (RNI), and optimizer overhead (OO) to compare the multiobjective solutions, as shown in Table 8 [5, 35].

#### 4.2.1. SSM

As it is desirable to find more Pareto-optimal solutions, it is also desirable to find the ones scattered uniformly over the Pareto frontier in order to provide a variety of compromise solutions to the decision makers. SSM denotes the distribution of the solutions along the Pareto front as shown below:

(22) $$\begin{array}{c} \text{SSM}=\frac{d_{f}+d_{l}+\sum_{i=1}^{N}\left|d_{i}-\overset{-}{d}\right|}{d_{f}+d_{l}+\left(N-1\right)\overset{-}{d}}, \end{array}$$

where* N* is the number of solutions along the Pareto front so there are (*N* − 1) consecutive distances,* d*_*i*_ is the distance (in objective space) between each solution, $\overset{-}{d}$ is the arithmetic mean of all* d*_*i*_ and* d*_*f*_ and* d*_*l*_, are the Euclidean distances between the extreme solutions and the boundary solutions of the obtained nondominated set. Thus, a low performance measure characterises an algorithm with good distribution capacity.

#### 4.2.2. RNI

This performance metric is defined as the ratio of nondominated individuals (RNI) for a given population *X*, as shown below:

(23) $$\begin{array}{c} \text{RNI}\left(X\right)=\frac{\text{nondom}\_\text{indiv}}{P}, \end{array}$$

where nondom_indiv is the number of nondominated individuals in population* X* and* P* is the size of population* X*. The value RNI = 1 means that all the individuals in the population are nondominated and RNI = 0 denotes the situation where none of the individuals in the population are nondominated. Since a population size of more than zero is often desired, there is at least one nondominated individual in the population within the range of 0 < RNI < 1.

#### 4.2.3. OO

The total number of evaluations and total CPU times may be used to test the algorithm. This is useful in indicating how much time an optimization or simulated evolution process would take in real world and to indicate the amount of program overhead as a result of the optimisation manipulations such as those by evolutionary algorithm operators, as shown in (24):

(24) $$\begin{array}{c} \text{OO}=\frac{T_{\text{Total}}-T_{\text{PFP}}}{T_{\text{PFP}}}, \end{array}$$

where *T*_Total_ is the total time taken and *T*_PFP_ is the time taken for pure function evaluations. Thus, a value of zero indicates that an algorithm is efficient and does not have any overhead. However, this is an ideal case and is not practically reachable.

Kruskal-Wallis testing (KW test) is an ANOVA test method that uses levels to test whether the above independent population allocations are the same. The statistics are as shown in below [32]:

(25) $$\begin{array}{c} K=\frac{12}{n\left(n+1\right)}\left(\overset{c}{\underset{i=1}{\sum }}\frac{R_{i}^{2}}{n_{i}}\right)-3\left(n+1\right). \end{array}$$

In (25), *n* is the total number of samples, *c* is the number of sample groups, *n*_*i*_ is the number of samples in the *i*th group, *i* = 1,…, *c*, and *R*_*i*_ is the addition of the levels of samples in* i*th group. When *H*_0_ is true, statistic* K* is the chi-square distribution of the degree of freedom *c* − 1. If *K* > *χ*_*c*−1,*α*_^2^, then *H*_0_ is rejected conversely, and *H*_0_ is accepted. In this paper,* K* is 8.68 > *χ*_3,0.05_^2^ = 7.81; therefore, the 4 kinds of algorithms have significantly different allocations.

Regarding the mutually nondominated solutions to the external registers by DPSO and PSO, whether each newly found solution can be included in the external register is determined by the relationship between the solution and the nondominated solution of the external register. If the external register size is not limited, there will be four possibilities [12], as described below.

1. For the first generation of evolution, there is no particle in the external register; thus, the newly found solution can be directly input into the external register.
2. If the newly found solution is dominated by the nondominated solution of the external register, the new solution will be deleted and the external register remains the same.
3. If the newly found solution and the nondominated solution of the external register are in a nondominated relationship, the new solution will be included in the external register.
4. If the newly found solution dominates the solution in the external register, then the dominated solution in the external register will be deleted and the new solution will be included in the external register.

The above four situations describe the pairwise comparison of newly found solutions and the solutions in the external registers. As the iterations continue, there will be increasing numbers of solutions in the external registers; thus, there will be more than one newly found solution. Therefore, in the case of a multiple-multiple particle relationship, they will be compared in pairs, and the external register will be updated accordingly.

**Figure 3:**
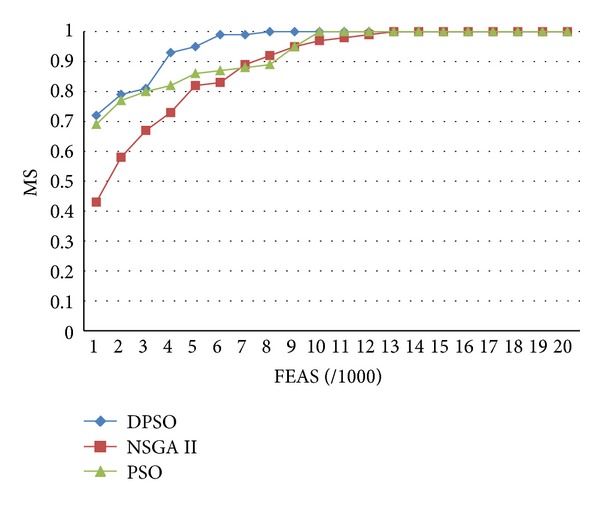
Convergence of 3 algorithms.

The MS convergence curve suggests that the convergence of three algorithms is similar. However, the convergence of the proposed algorithm is superior to the remaining two algorithms, as shown in Figure 3.

**Figure 4:**
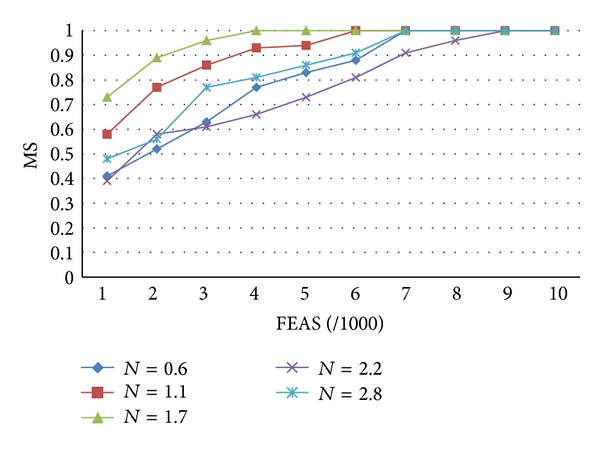
Comparison of *N* of different disturbance mechanisms.

Regarding the comparison of the disturbance mechanism, as described in Section 3.4.1, when the DR mechanism's* N* is 1.7, MS can have better convergence effects, as shown in Figure 4.

**Figure 5:**
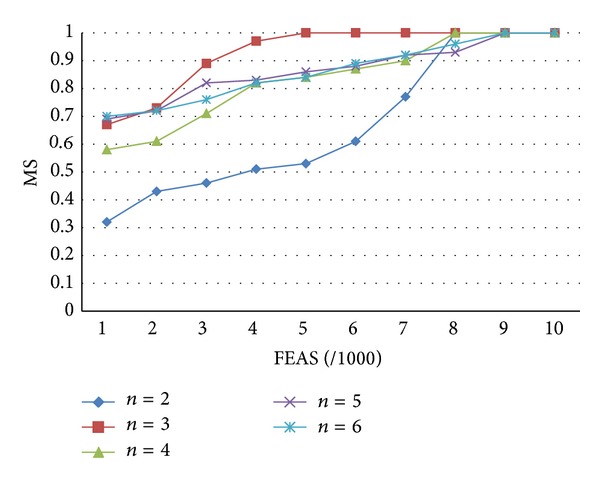
Comparison of number of competition items of tournament selection.

For the comparison of competing items in tournament selection, each experiment is performed 30 times.  Figure 5 shows the comparison of the competition items of tournament selection in the case of candidates 2 to 6. As seen, better convergence effect can be achieved when the number of race candidates (*n*) is 3.

## 5. Conclusions

This study established customer demand-oriented DPSO, classified products using different classification methods and evaluated the different inventory target performances. For enterprises with products of small quality and great variety, such as stationery discount stores, they can make appropriate inventory decisions in response to different customer demands using the proposed method. According to the research findings, although the clustering results of the proposed DPSO algorithm are superior to the results of the single-objective method, the ranking of the different results using the multiobjective method, as compared to individual objectives, is second, as shown in Table 4. Figures 3–5 compare DPSO, PSO, and NSGA-II, and show that the selection of the global optimal solution using a disturbance mechanism and tournament selection (DPSO) can have a better convergence effect. In the future, the proposed inventory decision processes may be applied in inventory classification decision making processes for similar types of business.

## Figures and Tables

**Table 1:** Comparison of single-objective target values obtained using various clustering methods.

	Clustering rule			
	DPSO	PSO	ABC	G
Total cost (ranking)	2635849.67 (1)	2958430.22 (2)	3279856.38 (3)	4912785.69 (4)
Backorder rate (ranking)	86% (1)	76% (2)	51% (3)	32% (4)
Demand relevance (ranking)	36.18 (1)	33.29 (2)	22.13 (3)	2.3 (4)
Inventory turnover rate (ranking)	19.83 (1)	16.32 (2)	12.22 (3)	3.23 (4)

**Table 2:** Analysis of the difference in cost using the DPSO clustering method and other clustering methods.

	Clustering rule				Cost savings	Saving percentage
	DPSO	PSO	ABC	G		
DPSO : PSO	2635849.67	2958430.22	—	—	10.9%	10.9%
DPSO : ABC	2635849.67	—	3279856.38	—	644,006.71	19.64%
DPSO : G	2635849.67	—	—	4912785.69	2,276,936.02	46.35%

**Table 3:** Difference analysis of the multiobjective target value and single-objective target value.

	Target value			
	Total cost	Backorder rate	Demand relevance	Inventory turnover rate
Single-objective	2635849.67	0.86	36.18	19.83
Multiobjective	2896386.23	0.87	34.29	18.11
Difference	260536.56	0.01	−1.89	−1.72
Percentage difference	9%	1.15%	−5.51%	−9.5%

**Table 4:** Difference analysis of the clustering results and target values of other target equations.

	Target value (percentage difference and ranking)			
	Total cost	Backorder rate	Demand relevance	Inventory turnover rate
Total cost	2,635,849.67 (9%, 1)	0.79 (10.13%, 4)	22.38% (34.73%, 4)	12.35% (31.81%, 4)
Backorder rate	3,623,541.22 (−25.11%, 4)	0.86 (1.15%, 2)	19.26% (43.83%, 5)	10.19% (43.13%, 5)
Demand relevance	3,689,236.25 (−27.37%, 5)	0.82 (5.75%, 3)	36.18% (−5.51%, 1)	13.68% (24.46%, 3)
Inventory turnover rate	2,965,872.37 (−2.4%, 3)	0.76 (12.64%, 5)	33.29% (2.92%, 3)	19.83% (9.5%, 1)
Multiobjective	2,896,386.23 (0%, 2)	0.87 (0%, 1)	34.29% (0%, 2)	18.11% (0%, 2)

**Table 5:** Settings of different multiobjective optimisation algorithm parameters.

	DPSO	PSO	NSGA-II
Population size	120	120	120
Size of external repository	Not limited	Not limited	120
Cross-over rate	N/A	N/A	0.8
Jump mechanism Operation number	120	N/A	N/A
Mutation rate	N/A	N/A	0.2
Disturbance rate	0.16	N/A	N/A
FEAS	20,000	20,000	20,000

**Table 6:** Parameter setup of the PAES and NSHS.

PAES	NSHS
Archive size: 120	*K*: 120
Number of regions: 12	(Harmony memory considering rate, HMCR): 0.8
*P*_*M*_: 0.7	(Pitch adjusting rate, PAR): 0.03
*P*_*N*_: 0.2	(Random selection rate, RSR): 0.02
*I*_*P*_: 6,000	*I*_HS_: 1,000
FEAS: 10^5^	FEAS: 10^5^

**Table 7:** The algorithms of the NLE values (mean/minimum/standard deviation).

#	*R*	DPSO	NSGA II	PAES	NSHS
1	0.12	**36.28**/**16.38**/**7.86**	40.39/21.24/9.92	39.26/20.21/9.25	38.01/19.21/8.21
2	0.13	**33.21**/**14.21**/**6.32**	26.21/17.11/8.49	35.09/16.38/8.34	34.29/15.34/7.34
3	0.14	31.29/**15.31**/**2.69**	35.36/19.08/4.89	34.28/18.93/4.21	**30.98**/17.32/3.23
4	0.15	**30.62**/13.29/**1.08**	34.29/16.21/4.67	33.29/15.21/3.45	32.49/**12.27**/2.11
5	0.16	**28.39**/**12.38**/**2.38**	35.66/18.34/5.48	34.39/16.39/5.46	33.24/14.58/3.21
6	0.17	27.28/**11.26**/**1.02**	31.27/15.62/3.29	**26.38**/14.98/3.28	28.98/13.26/2.65
7	0.18	**26.33**/12.36/**3.69**	32.16/**11.73**/6.88	31.29/15.11/6.78	29.34/14.21/4.01
8	0.19	25.36/10.98/**1.12**	28.39/14.21/4.02	27.28/**9.29**/3.01	**23.72**/11.29/2.32
9	0.20	**24.39**/11.27/2.21	27.29/**10.66**/4.09	26.34/14.12/3.98	25.38/12.38/**1.6**
10	0.21	**22.18**/**9.86**/1.31	28.34/13.23/3.98	27.12/12.31/3.09	26.35/11.11/**0.98**
11	0.22	21.62/**12.39**/**3.21**	24.38/14.81/4.97	23.43/14.18/4.01	**20.37**/13.89/3.34
12	0.23	20.34/**10.12**/**1.69**	23.42/17.98/4.32	**19.36**/16.26/3.59	21.39/13.12/3.46
13	0.24	**19.38**/9.87/**1.89**	22.31/12.36/5.01	21.31/11.36/4.98	20.98/**8.98**/4.23
14	0.25	18.21/**8.68**/**3.68**	21.36/14.52/5.43	20.39/12.31/5.02	**17.87**/10.23/4.98
15	0.26	**16.24**/**9.32**/**1.01**	20.98/11.5/4.09	19.36/10.93/3.09	17.21/9.87/2.48
16	0.27	**15.19**/**8.01**/**1.82**	18.16/13.6/3.98	17.38/12.18/3.08	16.39/9.96/2.36

**Table 8:** The comparison between the multiobjective solvers.

#	*R*	Indicator	DPSO	NSGA II	PAES	NSHS
1	0.12	SSM	**0.682**	0.703	0.701	0.693
		RNI	**0.987**	0.871	0.862	0.899
		OO	0.012	0.042	0.029	**0.011**

2	0.13	SSM	**0.589**	0.642	0.638	0.623
		RNI	**0.962**	0.893	0.908	0.912
		OO	**0.016**	0.033	0.022	0.029

3	0.14	SSM	0.732	0.792	0.758	**0.731**
		RNI	**0.891**	0.811	0.832	0.814
		OO	**0.023**	0.036	0.029	0.031

4	0.15	SSM	**0.693**	0.736	0.713	0.702
		RNI	**0.913**	0.872	0.899	0.909
		OO	0.024	0.052	**0.022**	0.041

5	0.16	SSM	**0.701**	0.762	0.788	0.789
		RNI	**0.983**	0.892	0.909	0.912
		OO	**0.019**	0.026	0.032	0.029

6	0.17	SSM	0.823	0.829	0.836	**0.813**
		RNI	**0.966**	0.959	0.96	0.961
		OO	0.018	0.029	0.024	**0.016**

7	0.18	SSM	**0.712**	0.748	0.721	0.736
		RNI	0.873	0.765	**0.893**	0.812
		OO	**0.036**	0.048	0.046	0.041

8	0.19	SSM	**0.612**	0.62	0.619	0.623
		RNI	**0.861**	0.812	0.822	0.834
		OO	**0.015**	0.031	0.022	0.019

9	0.20	SSM	**0.514**	0.521	0.534	0.526
		RNI	**0.985**	0.952	0.961	0.965
		OO	**0.019**	0.038	0.028	0.026

10	0.21	SSM	0.598	0.616	0.609	**0.591**
		RNI	**0.988**	0.955	0.963	0.979
		OO	0.026	0.043	**0.023**	0.032

11	0.22	SSM	**0.612**	0.618	0.622	0.621
		RNI	**0.892**	0.693	0.765	0.795
		OO	**0.016**	0.041	0.033	0.032

12	0.23	SSM	**0.636**	0.642	0.661	0.658
		RNI	**0.791**	0.723	0.764	0.754
		OO	**0.017**	0.031	0.022	0.021

13	0.24	SSM	**0.701**	0.802	0.711	0.737
		RNI	**0.898**	0.812	0.834	0.876
		OO	**0.019**	0.032	0.028	0.026

14	0.25	SSM	0.765	0.798	0.769	**0.713**
		RNI	0.911	0.812	**0.936**	0.856
		OO	**0.021**	0.036	0.032	0.028

15	0.26	SSM	0.512	0.522	0.548	**0.506**
		RNI	**0.928**	0.896	0.903	0.912
		OO	**0.018**	0.032	0.029	0.022

16	0.27	SSM	**0.493**	0.511	0.501	0.499
		RNI	0.916	0.886	**0.922**	0.909
		OO	**0.023**	0.038	0.036	0.034
